# Toxicity of engineered nanomaterials and their transformation products following wastewater treatment on A549 human lung epithelial cells

**DOI:** 10.1016/j.toxrep.2014.08.017

**Published:** 2014-09-21

**Authors:** Yanjun Ma, Subbiah Elankumaran, Linsey C. Marr, Eric P. Vejerano, Amy Pruden

**Affiliations:** a Department of Civil and Environmental Engineering, 418 Durham Hall, Virginia Tech, Blacksburg, VA 24061, United States; b Department of Biomedical Sciences & Pathobiology, Center for Molecular Medicine and Infectious Disease, Virginia-Maryland College of Veterinary Medicine, Virginia Tech, Blacksburg, VA 24061, United States

**Keywords:** Nanomaterials, Wastewater treatment, A549 lung epithelial cells, Cytotoxicity, Genotoxicity

## Abstract

Here we characterize the toxicity of environmentally-relevant forms of engineered nanomaterials (ENMs), which can transform during wastewater treatment and persist in aqueous effluents and biosolids. In an aerosol exposure scenario, cytotoxicity and genotoxicity of effluents and biosolids from lab-scale sequencing batch reactors (SBRs) to A549 human lung epithelial cells were examined. The SBRs were dosed with nanoAg, nano zero-valent iron (NZVI), nanoTiO_2_ and nanoCeO_2_ at sequentially increasing concentrations from 0.1 to 20 mg/L. Toxicities were compared to outputs from SBRs dosed with ionic/bulk analogs, undosed SBRs, and pristine ENMs. Pristine nanoAg and NZVI showed significant cytotoxicity to A549 cells in a dose-dependent manner from 1 to 67 μg/mL, while nanoTiO_2_ and nanoCeO_2_ only exerted cytotoxicity at 67 μg/mL. Only nanoAg induced a genotoxic response, at 9, 33 and 53 μg/mL. However, no significant cytotoxic or genotoxic effects of the SBR effluents or biosolids containing nanomaterials were observed.

## Highlights

• Toxicity of nanomaterials can be altered following wastewater treatment process.
• Pristine nanomaterials showed different extent of cytotoxicity and genotoxicity to A549 cells.
• No cytotoxic or genotoxic effects to A549 cells were observed for nanomaterials following wastewater treatment.

## Graphical abstract

**Figure:**
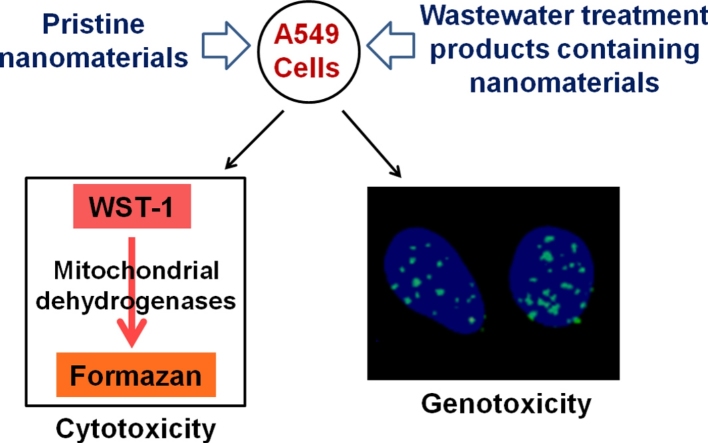

## 1 Introduction

The concern that engineered nanomaterials (ENMs) may have adverse effects on human health is increasing as application of nanotechnology in consumer products expands [1], [2]. Humans can be exposed to ENMs in the workplace and during use of nano-products, and also through contact with water, soil, or air to which ENMs may have been released [3]. Comprehensive risk assessment of ENMs requires characterization of the toxicity of ENMs under a wide range of exposure conditions, including environmental routes. Cytotoxicity and genotoxicity of common metal and carbon nanomaterials, such as nanoAg, nanoTiO_2_, and carbon nanotubes have been widely studied in human lung, dermal, and visceral cells [4], [5], [6], [7]. However, in assessing the risks of ENMs released into the natural environment, available data on the toxicity of environmentally-relevant forms of ENMs is lacking [8], [9]. Extrapolation of toxicity data based on testing of pristine ENMs may not be appropriate because ENMs are highly reactive by nature and can be chemically, physically and biologically transformed in the environment, potentially altering their toxicity [10], [11].

Wastewater treatment plants (WWTPs) are a critical route of ENM receipt and release into the natural environment [12], [13], [14]. The complex wastewater matrix is likely to favor transformation of ENMs. A small portion of ENMs will remain in the wastewater effluent, while the majority will associate with the sludge [15], [16] and eventually be disposed of by land-application, landfill, or incineration [12]. During the reuse of treated wastewater and land-application of waste sludge (biosolids), there is potential for humans to be exposed to transformed ENMs, especially through inhalation of aerosols generated [17], [18]. However, to the knowledge of the authors, impacts of human exposure to transformed ENMs following wastewater treatment have not previously been reported.

This study examined cytotoxicity and genotoxicity of effluents and biosolids from lab-scale sequencing batch reactors (SBRs) receiving ENMs to A549 human lung epithelial cells. The SBRs were dosed with nanoAg, nano zero-valent iron (NZVI), nanoTiO_2_ and nanoCeO_2_ at sequentially increasing concentrations from 0.1 to 20 mg/L. Toxicities were compared to outputs from SBRs dosed with ionic/bulk analogs (Ag^+^, Fe^2+^, bulkTiO_2_ and bulkCeO_2_), outputs from undosed SBRs, and pristine ENMs.

## 2 Materials and methods

### 2.1 Preparation of samples

NanoAg (52 ± 12 nm, prepared using citrate reduction method), NZVI (46 ± 10 nm, NANOFER 25S, Rajhrad, Czech Republic), nanoTiO_2_ (21 ± 12 nm, anatase nanopowder, Sigma–Aldrich, Saint Louis, MO), nanoCeO_2_ (33 ± 12 nm, Sigma–Aldrich) and their ionic/bulk analogs [Ag^+^ as AgNO_3_ (Fisher, Suwanee, GA), Fe^2+^ as FeSO_4_ (Fisher), bulkTiO_2_ (Sigma–Aldrich) and bulkCeO_2_ (Sigma–Aldrich)] were prepared/purchased and characterized as described in Ma et al. [35]. As previously reported (Ma et al. [35]), lab-scale nitrifying SBRs fed with synthetic wastewater were set up and operated at steady state under three conditions in duplicate: (1) SBRs dosed with nanoAg, NZVI, nanoTiO_2_, or nanoCeO_2_; (2) SBRs dosed with Ag^+^, Fe^2+^, bulkTiO_2_, or bulkCeO_2_; and (3) undosed SBRs. The dosing was initiated at 0.1 mg/L and sequentially increased to 1, 10 and 20 mg/L. The SBRs were actively nitrifying at the time of this study; further details on SBR performance are reported in Ma et al. [35]. Aqueous effluents and biosolids were sampled at the end of 20 mg/L dosing. In preparation for toxicity tests, samples from duplicate SBRs were combined and sterilized with concentrations of nanomaterials in SBR effluents and biosolids quantified by inductively coupled plasma mass spectrometry (ICP-MS), as described in the Supplementary Information.

### 2.2 Cell culture and treatment

A549 human lung alveolar epithelial cells were obtained from ATCC (#CCL-185, Manassas, VA). Cells were cultured in Dulbecco's Modified Eagle's Medium (DMEM, Thermo Scientific HyClone, Logan, UT) containing 10% heat inactivated fetal bovine serum (FBS, Atlanta Biologicals, Flowery Branch, GA) and 1% penicillin–streptomycin (Thermo Scientific HyClone, Logan, UT), and were maintained in a humidified incubator at 37 °C and 5% CO_2_. Cells were seeded at a density of 1 × 10^4^ in 100 μL culture medium in each well of 96-well plates. At 80% confluency, they were treated for 24 h either with pristine nanomaterials, ionic/bulk analogs, SBR aqueous effluents, or SBR biosolids as indicated. The exposure concentrations of the pristine materials (Table S1) varied from 1 to 67 μg/mL for the cytotoxicity assay to estimate IC_50_ values. Genotoxicity assays were carried out at key concentration values based on the results of the cytotoxicity assays (Table S1). The exposure concentrations of nanomaterials and ionic/bulk materials in SBR effluents and biosolids are shown in Table 1. All samples were diluted with the culture medium to target concentrations.

**Table 1:** Concentration of nanomaterials and ionic/bulk materials in SBR effluents and biosolids, and exposure concentration in cytotoxicity and genotoxicity assays.

	SBR effluents (μg/L)	Exposure concentration (μg/L)^a^	Biosolids (μg/g total solids)	Exposure concentration (μg/mL)^b^
NanoAg	28.43	9.48	2332	0.47
Ag^+^	43.67	14.56	2212	0.44
NZVI	7.10	2.37	38,327	7.67
Fe^2+^	4.70	1.57	48,061	9.61
NanoTiO_2_	0.95^c^	0.32	49,201	9.84
BulkTiO_2_	1.02^c^	0.34	67,649	13.53
NanoCeO_2_	0.25	0.08	22,360	4.47
BulkCeO_2_	0.86	0.29	26,454	5.29

a SBR effluents were diluted approximately 1:3 in the culture medium.

b SBR biosolids were exposed to A549 cells at 200 μg total solids/mL.

c Concentrations of nanoTiO_2_ and bulkTiO_2_ in SBR effluents were near the detection limit of Ti by ICP-MS and not significantly different from concentrations in the undosed SBR.

### 2.3 Cytotoxicity assay

Cell viability was measured using the WST-1 assay (Roche, Indianapolis, IN) based on quantification of mitochondrial activity as an indicator of cytotoxicity. In viable cells, the tetrazolium salt WST-1 is converted to soluble formazan dye by mitochondrial succinate-tetrazolium reductase, which can be quantified by absorbance. After the A549 cells were treated with the samples for 24 h, WST-1 reagent was added to each well at 1/10 volume of the medium. The absorbance was quantified after incubating at 37 °C for 3 h using a Tecan Safire^2^ Microplate Reader (Tecan US In., Research Triangle Pa, NC) at 440 nm with a reference wavelength of 660 nm. All treated A549 cells (a) were tested in triplicate in three independent experiments with three controls: (b) untreated A549 cells in culture medium; (c) samples in culture medium without A549 cells; and (d) culture medium only. In a single experiment, cell viability was calculated as percentage of the average absorbance derived from triplicate runs of treated cells relative to untreated control cells, with absorbances of corresponding controls subtracted out to address possible matrix interferences: fraction cell viability = [(a − c)/(b − d)].

### 2.4 Genotoxicity assay

DNA damage in A549 cells was detected using immunofluorescent labeling of γH2AX foci as described elsewhere [19]. At sites of DNA double strand breaks, H2AX, a minor nucleosomal histone protein, is rapidly phosphorylated and forms γH2AX [20]. The experimental and imaging procedures are provided in the Supplementary Information. Three independent experiments were conducted with at least 200 cells imaged in a single test. Untreated cells and cells treated with 100 μM H_2_O_2_ for 10 min were included as negative and positive controls, respectively. Images were analyzed using ImageJ 1.47 (http://rsbweb.nih.gov/ij/) with a macro designed to subtract background and count the number of foci within the defined nucleus masks.

### 2.5 Statistical analysis

The data were presented as mean ± standard deviation of three independent experiments. Student's *t* test or pairwise *t* test was conducted in R-2.8.1 (http://cran.r-project.org/bin/windows/base/old/2.8.1/) to determine statistical differences between samples at a significance level of 0.05 (*p* < 0.05).

## 3 Results and discussion

### 3.1 Toxicity of pristine nanomaterials

Based on the WST-1 assay, the viability of cells exposed to nanoAg, Ag^+^, NZVI and Fe^2+^ for 24 h decreased significantly (*p* < 0.05) in a dose-dependent manner (Fig. S1). The IC_50_ values for these materials were estimated to be 53 ± 2, 21 ± 0.1, 38 ± 2 and 55 ± 2 μg/mL, respectively. In particular, the IC_50_ of Ag^+^ was significantly lower than that of nanoAg (*p* = 5 × 10^−4^), a result that is in agreement with other studies using A549 cell targets [21], [4]. By contrast, NZVI was more toxic than its ionic analog, Fe^2+^ (*p* = 6 × 10^−4^). In another study, the cytotoxicty of NZVI (synthesized through reduction of FeCl_3_ by NaBH_4_ and coated with Pd) to human bronchial epithelial cells 16HBE14o was not significantly different from Fe^2+^ [22]. Differences between the studies could relate to differences in NZVI coatings (manufacturer in present study reports biodegradable organic and inorganic stabilizers) or the different cells used in the assays (A549 *versus* 16HBE14o). The viability of cells exposed to nanoTiO_2_, nanoCeO_2_ and their bulk analogs only decreased at a concentration of 67 μg/mL, by 10–20% (Fig. S1, *p* < 0.05). But in a few other studies, no significant cytotoxicity of nanoTiO_2_ and nanoCeO_2_ to A549 cells was observed, even at concentrations up to 100 μg/mL [23], [5], [24].

Only cells that were exposed to 33 and 53 μg/mL nanoAg and 21 μg/mL Ag^+^ showed significantly higher numbers of γH2AX foci per cell (Fig. S2A) and greater percentages of cells containing γH2AX foci (Fig. S2B) (*p* < 0.05) relative to untreated control cells, suggesting genotoxicity resulting from DNA double strand breaks. The number of γH2AX foci per cell exposed to 9 μg/mL nanoAg was not significantly different from that of the control cells (Fig. S2A, *p* = 0.12), but the percentage of cells containing γH2AX foci was significantly higher than for the control cells (Fig. S2B, *p* = 0.03). Although IC_50_ concentrations of NZVI and Fe^2+^ (38 and 55 μg/mL, respectively), and nanoTiO_2_, nanoCeO_2_ and the bulk analogs at 67 μg/mL induced significant cytotoxicity to A549 cells, the number of γH2AX foci were not significantly different in cells treated with these materials (Fig. S2, *p* > 0.05). To the authors’ knowledge, genotoxicity of NZVI to human cells has not been investigated previously. Genotoxicities have been observed in A549 cells at concentrations of 2.5–15 μg/mL of nanoAg by ^32^P post-labeling of DNA adducts [4], 10–50 μg/mL of nanoTiO_2_ by cytokinesis block micronucleus assay [25], and 0.5–100 μg/mL of nanoCeO_2_ by alkaline comet assay [23]. However, in this study, the genotoxicity of nanoAg could be demonstrated, but not nanoTiO_2_ or nanoCeO_2_ at 67 μg/mL. The distinct outcome of the nanoTiO_2_ in the present study could be attributed to the different genotoxicity assays employed. With respect to nanoCeO_2_, the prior study [23] utilized lab-synthesized nanoCeO_2_ with a size range of 16–22 nm, whereas the present study utilized commercial nanoCeO_2_ with an average particle size of 33 ± 12 nm.

### 3.2 Toxicity of SBR effluents and biosolids

**Fig. 1:**
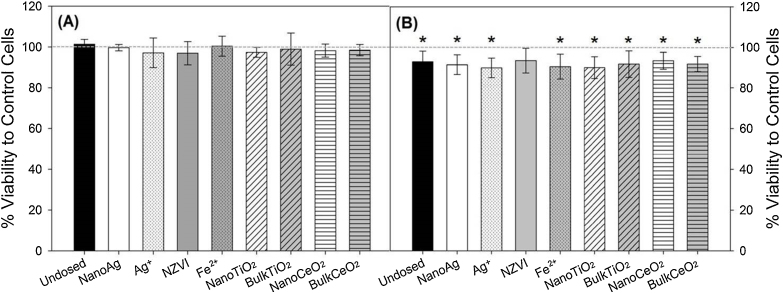
Characteristic cytotoxicity of A549 cells exposed to (A) wastewater effluents and (B) biosolids from undosed SBR, and SBRs dosed with nanoAg, Ag^+^, NZVI, Fe^2+^, nanoTiO_2_, bulkTiO_2_, nanoCeO_2_, and bulkCeO_2_ for 24 h by WST-1 assay. Exposure concentrations are shown in Table 1. Error bars represent standard deviations of three independent experiments. “*” indicates significant decrease of viability compared with untreated control cells (*p* < 0.05).

**Fig. 2:**
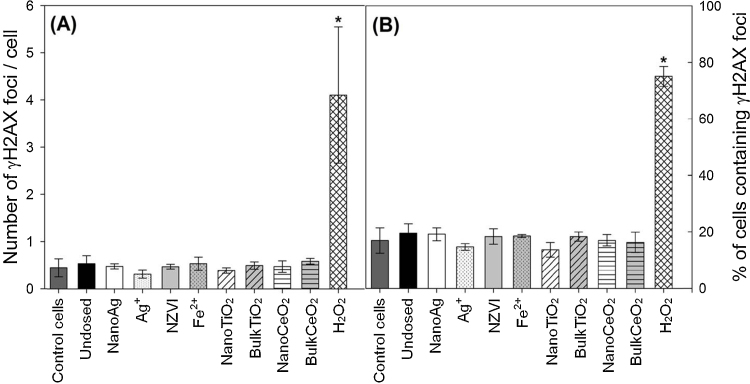
γH2AX foci in untreated control A549 cells; cells treated with wastewater effluents from undosed SBR and SBRs dosed with nanoAg, Ag^+^, NZVI, Fe^2+^, nanoTiO_2_, bulkTiO_2_, nanoCeO_2_, and bulkCeO_2_ for 24 h; and cells treated with 100 μM H_2_O_2_ for 10 min. Exposure concentrations of materials are shown in Table 1. Data are presented as (A) number of γH2AX foci per cell and (B) percentage of cells containing γH2AX foci. Error bars represent standard deviations of three independent experiments. “*” indicates significant difference compared with untreated control cells (*p* < 0.05).

Based on a previous study (Ma et al. [35]), >99% of nanomaterials and ionic/bulk analogs dosed into the SBRs partitioned into the sludge relative to the influent concentrations. Concentrations of nanomaterials in SBR aqueous effluents and biosolids, as well as exposed to A549 cells, are shown in Table 1. No significant decrease of cell viability or induction of γH2AX foci were observed in A549 cells exposed to SBR effluents (Fig. 1, Fig. 2, *p* > 0.05). The exposure concentration of nanoAg in this study (9.48 μg/L) was much higher than predicted concentrations in WWTP effluents (<0.5 μg/L) [12], [13], [14], while the exposure concentrations of NZVI (2.37 μg/L), nanoTiO_2_ (0.32 μg/L), and nanoCeO_2_ (0.08 μg/L) were within or lower than the lower bound concentrations predicted (0.7–20 μg/L for NZVI [14], 1–70 μg/L for nanoTiO_2_ [12], [13], [14], and 0.5 × 10^−4^ to 2 μg/L for nanoCeO_2_ [13], [14]. Results of this study indicated limited toxicity of nanoAg in wastewater effluents at higher than environmentally-relevant concentrations to A549 cells, while the effects of higher concentrations of NZVI, nanoTiO_2_ and nanoCeO_2_ remain to be determined.

The concentrations of nanomaterials in biosolids in this study (>2000 μg/g dry mass, Table 1) were significantly higher than concentrations predicted in biosolids from WWTPs (<1000 μg/g dry mass) [12], [13], [14]. Moreover, 200 μg total solids/mL represented a high exposure dose relative to other studies of effects of aerosolized biosolids to human lung cells [26]. The viability of cells exposed to biosolids at 200 μg total solids/mL decreased by 7–10% relative to untreated control cells (*p* < 0.05), except for biosolids containing NZVI (*p* = 0.08) (Fig. 1B). But, there was no significant difference between cells exposed to biosolids from undosed *versus* dosed SBRs (*p* > 0.05), indicating that the decrease in cell viability was not likely due to the nanomaterials or ionic/bulk materials, but probably instead to the high concentration of total solids. Cytotoxicity of biosolids was also examined, at 50 and 100 μg total solids/mL, and no significant effects were observed relative to control cells (*p* > 0.05, Fig. S3).

**Fig. 3:**
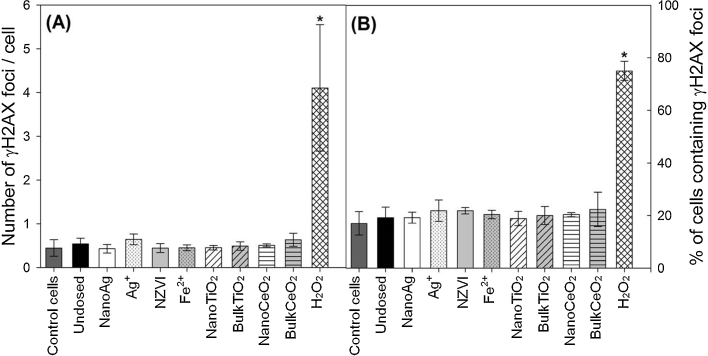
γH2AX foci in untreated control A549 cells; cells treated with biosolids from undosed SBR and SBRs dosed with nanoAg, Ag^+^, NZVI, Fe^2+^, nanoTiO_2_, bulkTiO_2_, nanoCeO_2_, and bulkCeO_2_ for 24 h; and cells treated with 100 μM H_2_O_2_ for 10 min. Exposure concentration of materials were shown in Table 1. Data was presented as (A) number of γH2AX foci per cell and (B) percentage of cells containing γH2AX foci. Error bars represent standard deviations of three independent experiments. “*” indicates significant difference compared with untreated control cells (*p* < 0.05).

Genotoxicity of biosolids was examined at 200 μg total solids/mL. However, no significant differences were observed in terms of the number of γH2AX foci per cell or in the percentage of cells containing γH2AX foci in cells treated with biosolids relative to untreated control cells (Fig. 3, *p* > 0.05), suggesting little or no DNA damage to A549 cells at concentrations exceeding most likely aerosol exposure levels.

Uptake of pristine nanoAg, nanoTiO_2_, and nanoCeO_2_ by human cells [21], [5], [24], [27], and NZVI by mammalian nerve cells [28] has been observed in previous studies, and the most commonly identified mechanism of toxicity was the generation of reactive oxygen species, which induced oxidative stress [27], [4], [22], [25]. Release of Ag^+^ was considered another potential cause of nanoAg toxicity [29]. Based on TEM-EDS mapping carried out in a previous study of the SBR biosolids (Ma et al. [35]), while a large portion of nanoAg remained dispersed, it mainly formed Ag–S complexes. Sulfidation has been reported to reduce toxicity of nanoAg to microbes, aquatic and terrestrial eukaryotic organisms due to low solubility of Ag–S complexes [30], [31]. Similarly, transformation of nanoAg in this study may limit its reactivity and result in little toxicity of SBR biosolids to A549 cells. The majority of NZVI, nanoTiO_2_ and nanoCeO_2_ were aggregated, but not chemically modified. Epithelial cells are impervious to aggregated nanomaterials by diffusion or macropinocytosis [32]. Therefore, the absence of cytotoxicity and genotoxicity of SBR biosolids in this study could be attributed to the inability of aggregated NZVI, nanoTiO_2_, and nanoCeO_2_ to enter cells. However, the size of the nanomaterial aggregate can affect its physiological distribution and kinetics, cellular distribution (for example within the draining lymph node for an aerosolized particle), cellular uptake, and intracellular processing pathways [33]. Also, it is difficult to ascertain the fate of aerosolized nanomaterials from biosolids in the respiratory tract as particles can diffuse and convert during interstitial transport depending on their size [34]. Thus, future work may consider the toxicity under *ex vivo* and *in vivo* conditions.

## Conflict of interest

The authors declare no conflicts of interest.

## Transparency document

Transparency document

## References

[1] Papp T., Schiffmann D., Weiss D., Castranova V., Vallyathan V., Rahman Q. (2008). Human health implications of nanomaterial exposure. Nanotoxicology.

[2] Xia T., Li N., Nel A.E. (2009). Potential health impact of nanoparticles. Annu. Rev. Publ. Health.

[3] Colvin V.L. (2003). The potential environmental impact of engineered nanomaterials. Nat. Biotechnol..

[4] Foldbjerg R., Dang D.A., Autrup H. (2011). Cytotoxicity and genotoxicity of silver nanoparticles in the human lung cancer cell line, A549. Arch. Toxicol..

[5] Lankoff A., Sandberg W.J., Wegierek-Ciuk A., Lisowska H., Refsnes M., Sartowska B., Schwarze P.E., Meczynska-Wielgosz S., Wojewodzka M., Kruszewski M. (2012). The effect of agglomeration state of silver and titanium dioxide nanoparticles on cellular response of HepG2, A549 and THP-1 cells. Toxicol. Lett..

[6] Lewinski N., Colvin V., Drezek R. (2008). Cytotoxicity of nanoparticles. Small.

[7] Lindberg H.K., Falck G.C.M., Suhonen S., Vippola M., Vanhala E., Catalan J., Savolainen K., Norppa H. (2009). Genotoxicity of nanomaterials: DNA damage and micronuclei induced by carbon nanotubes and graphite nanofibres in human bronchial epithelial cells in vitro. Toxicol. Lett..

[8] Blaser S.A., Scheringer M., MacLeod M., Hungerbuhler K. (2008). Estimation of cumulative aquatic exposure and risk due to silver: contribution of nano-functionalized plastics and textiles. Sci. Total Environ..

[9] Klaine S.J., Koelmans A.A., Horne N., Carley S., Handy R.D., Kapustka L., Nowack B., von der Kammer F. (2012). Paradigms to assess the environmental impact of manufactured nanomaterials. Environ. Toxicol. Chem..

[10] Nowack B., Ranville J.F., Diamond S., Gallego-Urrea J.A., Metcalfe C., Rose J., Horne N., Koelmans A.A., Klaine S.J. (2012). Potential scenarios for nanomaterial release and subsequent alteration in the environment. Environ. Toxicol. Chem..

[11] Wiesner M.R., Lowry G.V., Alvarez P., Dionysiou D., Biswas P. (2006). Assessing the risks of manufactured nanomaterials. Environ. Sci. Technol..

[12] Gottschalk F., Sonderer T., Scholz R.W., Nowack B. (2009). Modeled environmental concentrations of engineered nanomaterials (TiO_2_, ZnO, Ag, CNT, Fullerenes) for different regions. Environ. Sci. Technol..

[13] Gottschalk F., Sun T.Y., Nowack B. (2013). Environmental concentrations of engineered nanomaterials: review of modeling and analytical studies. Environ. Pollut..

[14] Keller A.A., Lazareva A. (2014). Predicted releases of engineered nanomaterials: from global to regional to local. Environ. Sci. Technol. Lett..

[15] Kiser M.A., Westerhoff P., Benn T., Wang Y., Perez-Rivera J., Hristovski K. (2009). Titanium nanomaterial removal and release from wastewater treatment plants. Environ. Sci. Technol..

[16] Limbach L.K., Bereiter R., Mueller E., Krebs R., Gaelli R., Stark W.J. (2008). Removal of oxide nanoparticles in a model wastewater treatment plant: influence of agglomeration and surfactants on clearing efficiency. Environ. Sci. Technol..

[17] Bausum H.T., Schaub S.A., Kenyon K.F., Small M.J. (1982). Comparison of coliphage and bacterial aerosols at a wastewater spray irrigation site. Appl. Environ. Microbiol..

[18] Paez-Rubio T., Ramarui A., Sommer J., Xin H., Anderson J., Peccia J. (2007). Emission rates and characterization of aerosols produced during the spreading of dewatered class B biosolids. Environ. Sci. Technol..

[19] Yu Y.K., Zhu W., Diao H.L., Zhou C.X., Chen F.Q.F., Yang J. (2006). A comparative study of using comet assay and gamma H2AX foci formation in the detection of N-methyl-N’-nitro-N-nitrosoguanidine-induced DNA damage. Toxicol. In Vitro.

[20] Rogakou E.P., Pilch D.R., Orr A.H., Ivanova V.S., Bonner W.M. (1998). DNA double-stranded breaks induce histone H2AX phosphorylation on serine 139. J. Biol. Chem..

[21] Cronholm P., Karlsson H.L., Hedberg J., Lowe T.A., Winnberg L., Elihn K., Wallinder I.O., Moller L. (2013). Intracellular uptake and toxicity of ag and cuo nanoparticles: a comparison between nanoparticles and their corresponding metal ions. Small.

[22] Keenan C.R., Goth-Goldstein R., Lucas D., Sedlak D.L. (2009). Oxidative stress induced by zero-valent iron nanoparticles and Fe(II) in human bronchial epithelial cells. Environ. Sci. Technol..

[23] De Marzi L., Monaco A., De Lapuente J., Ramos D., Borras M., Di Gioacchino M., Santucci S., Poma A. (2013). Cytotoxicity and genotoxicity of ceria nanoparticles on different cell lines in vitro. Int. J. Mol. Sci..

[24] Moschini E., Gualtieri M., Colombo M., Fascio U., Camatini M., Mantecca P. (2013). The modality of cell–particle interactions drives the toxicity of nanosized CuO and TiO_2_ in human alveolar epithelial cells. Toxicol. Lett..

[25] Srivastava R.K., Rahman Q., Kashyap M.P., Singh A.K., Jain G., Jahan S., Lohani M., Lantow M., Pant A.B. (2013). Nano-titanium dioxide induces genotoxicity and apoptosis in human lung cancer cell line, A549. Human Exp. Toxicol..

[26] Viau E., Levi-Schaffer F., Peccia J. (2010). Respiratory toxicity and inflammatory response in human bronchial epithelial cells exposed to biosolids, animal manure, and agricultural soil particulate matter. Environ. Sci. Technol..

[27] Eom H.J., Choi J. (2009). Oxidative stress of CeO_2_ nanoparticles via p38-Nrf-2 signaling pathway in human bronchial epithelial cell, Beas-2B. Toxicol. Lett..

[28] Phenrat T., Long T.C., Lowry G.V., Veronesi B. (2009). Partial oxidation (aging) and surface modification decrease the toxicity of nanosized zerovalent iron. Environ. Sci. Technol..

[29] AshaRani P.V., Mun G.L.K., Hande M.P., Valiyaveettil S. (2009). Cytotoxicity and genotoxicity of silver nanoparticles in human cells. ACS Nano.

[30] Levard C., Hotze E.M., Colman B.P., Dale A.L., Truong L., Yang X.Y., Bone A.J., Brown G.E., Tanguay R.L., Di Giulio R.T., Bernhardt E.S., Meyer J.N., Wiesner M.R., Lowry G.V. (2013). Sulfidation of silver nanoparticles: natural antidote to their toxicity. Environ. Sci. Technol..

[31] Reinsch B.C., Levard C., Li Z., Ma R., Wise A., Gregory K.B., Brown G.E., Lowry G.V. (2012). Sulfidation of silver nanoparticles decreases *Escherichia coli* growth inhibition. Environ. Sci. Technol..

[32] Albanese A., Chan W.C.W. (2011). Effect of gold nanoparticle aggregation on cell uptake and toxicity. ACS Nano.

[33] Oberdorster G., Ferin J., Lehnert B.E. (1994). Correlation between particle-size, in-vivo particle persistence, and lung injury. Environ. Health Perspect..

[34] Semmler-Behnke M., Takenaka S., Fertsch S., Wenk A., Seitz J., Mayer P., Oberdorster G., Kreyling W.G. (2007). Efficient elimination of inhaled nanoparticles from the alveolar region: evidence for interstitial uptake and subsequent re-entrainment onto airway epithelium. Environ. Health Perspect..

[35] Ma Y., Metch J.W., Vejerano E.P., Miller I.J., Leon E.C., Marr L.C., Vikesland P.J., Pruden A. (2014). Microbial community response of nitrifying sequencing batch reactors to silver, zero-valent iron, titanium dioxide and cerium dioxide nanomaterials, Water Res..

