# Supplementary material for: Toxicity of engineered nanomaterials and their transformation products following wastewater treatment on A549 human lung epithelial cells
Source: Toxicol Rep. 2014 Sep 21;1:871–6. doi: 10.1016/j.toxrep.2014.08.017 (PMC5598501; doi:10.1016/j.toxrep.2014.08.017)
Supplement: Supplementary file 2 [file mmc1.docx]

Toxicity of Engineered Nanomaterials and their Transformation Products following Wastewater Treatment on A549 Human Lung Epithelial Cells

Yanjun Ma, Subbiah Elankumaran, Linsey C. Marr, Eric P. Vejerano, Amy Pruden*

^*^ Corresponding author phone: (540)231-3980; fax: (540)231-7916; e-mail: [apruden@vt.edu](mailto:apruden@vt.edu). Current address: Via Department of Civil and Environmental Engineering, 418 Durham Hall, Virginia Tech, Blacksburg, VA 24061.

**Supplementary information**

**1. Preparation of SBR effluents and biosolids for toxicity assays**

Wastewater effluents from SBRs were filtered through 0.45 µm mixed-cellulose ester membrane filters (EMD Millipore, Billerica, MA) to remove bacteria, and biosolids were freeze-dried (FreeZone Plus 4.5, Labconco, Kansas City, MO). A subsample of 1.6 mg of freeze-dried biosolids was sterilized with 0.5 mL 100% ethanol and air-dried in a fume hood. The ethanol-treated biosolids were suspended in 1.6 mL 0.1% PBS-Tween solution by sonication for 30 min.

Concentration of nanomaterials and bulk/ionic materials in wastewater effluents and biosolids were analyzed using ICP-MS (Thermo Scientific, Tewksbury, MA) to quantify Ag, Fe, Ti and Ce. Aqueous effluents were filtered through 0.45 µm mixed-cellulose ester (MCE) membrane filters prior to analysis. Activated sludge containing nanoAg was digested with 1:3 hydrochloric to nitric acid (v/v), and activated sludge containing NZVI, nanoTiO_2_, and nanoCeO_2_ was digested by 1:1 sulfuric to nitric acid (v/v). The digested samples were diluted and filtered through 0.45 µm MCE membrane filters for ICP-MS.

**2. Immunofluorescent labeling of γH2AX foci**

A549 cells were exposed to samples for 24 h, followed by washing with PBS and fixation in 4% paraformaldehyde for 15 min and permeabilization in 0.25% Triton X-100 for 15 min. After being blocked with 1% bovine serum albumin in PBS for 1 h, cells were incubated with a rabbit polyclonal anti-γH2AX antibody (1:500; Santa Cruz Biotechnology Inc., Paso Robles, CA) for 1 h. Following washing with PBS 3x, the cells were incubated with Alexa Fluor 488 Goat Anti-Rabbit IgG (H+L) Antibody (1:500, Life Technologies Corporation, Grand Island, NY) and Hoechst 33342 (10 µg/mL, Life Technologies Corporation, Grand Island, NY) for 1 h. After washing the cells three times with PBS, fluorescence was visualized under 40× objective of an EcLipse TS 100 fluorescent microscopy (Nikon, Melville, NY). γH2AX foci emitted green fluorescent and the nuclei were stained blue with Hoechst 33342.

**3. Supporting tables and figures**

**Table S1. Exposure concentration of pristine nanomaterials and ionic/bulk materials to A549 cells**

| **Assay** | **Exposure concentration** | | | | | | | |
| --- | --- | --- | --- | --- | --- | --- | --- | --- |
|  | NanoAg | Ag^+^ | NZVI | Fe^2+^ | Nano  TiO_2_ | Bulk  TiO_2_ | Nano  CeO_2_ | Bulk  CeO_2_ |
| **Cytotoxicity**  (µg/mL) | 1, 9, 33, 50, 60 and 67 | | | | | | | |
| **Genotoxicity***  (µg/mL) | 9,33,53 | 21 | 38 | 55 | 67 | 67 | 67 | 67 |

*Genotoxicity of nanoAg, Ag^+^, NZVI and Fe^2+^ was examined at IC_50_ obtained from cytotoxicity assay (53, 21, 38 and 55 µg/mL, respectively); nanoAg was also examined at 9 and 33 µg/mL because significant genotoxicity was observed at 53 µg/mL; nanoTiO_2_, bulkTiO_2_, nanoCeO_2_ and bulkCeO_2_ were examined at 67 µg/mL, because cytotoxicity was only observed at 67 µg/mL.


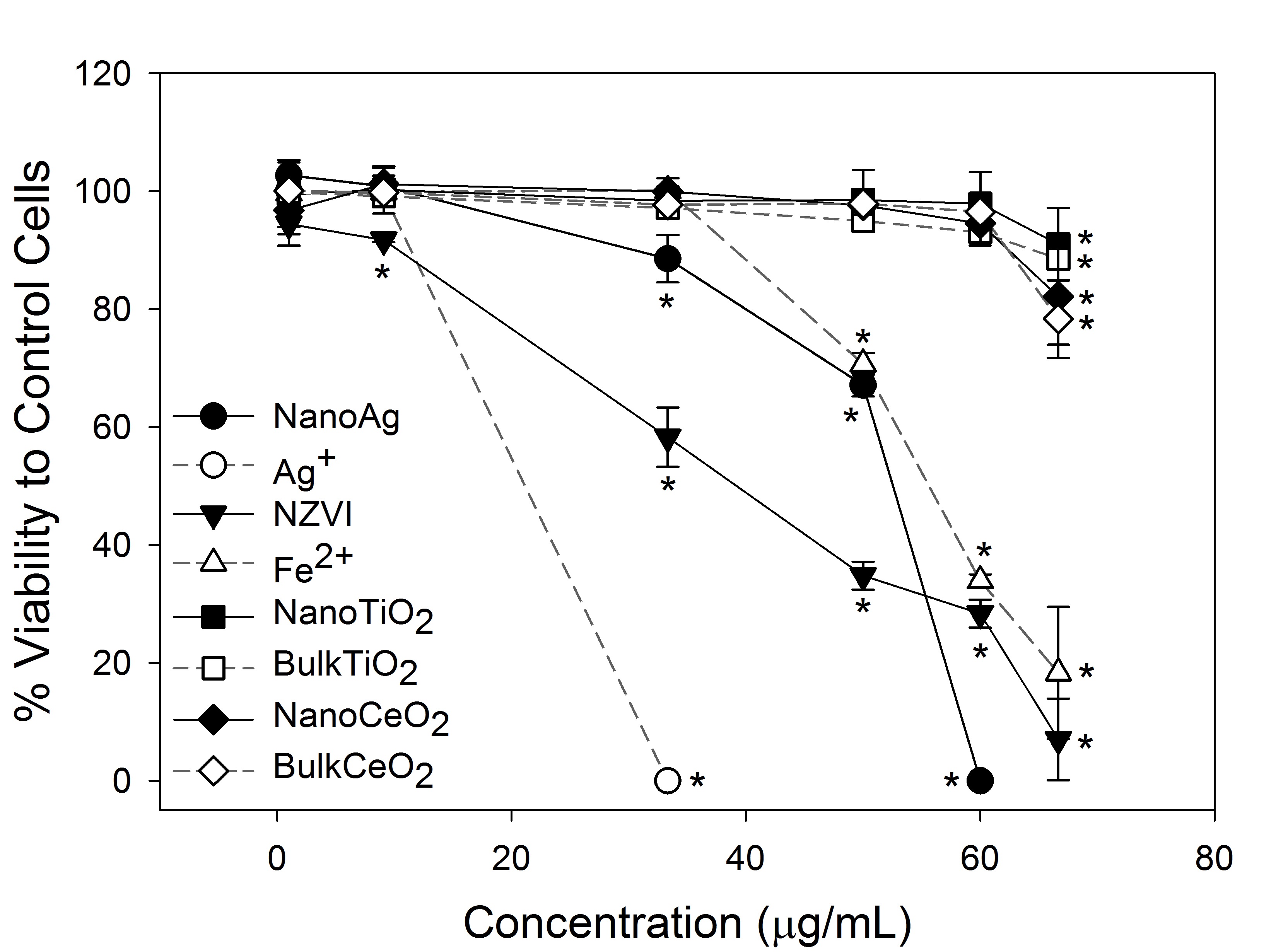


**Fig. S1**. Characteristic cytotoxicity of A549 cells exposed to nanoAg, Ag^+^, NZVI, Fe^2+^, nanoTiO_2_, bulkTiO_2_, nanoCeO_2_, and bulkCeO_2_ for 24 h by WST-1 assay. Six concentrations were tested: 1, 9, 33, 50, 60, and 67 µg/mL. Error bars represent standard deviations of three independent experiments. “*” indicates significant decrease compared with untreated control cells (p < 0.05).


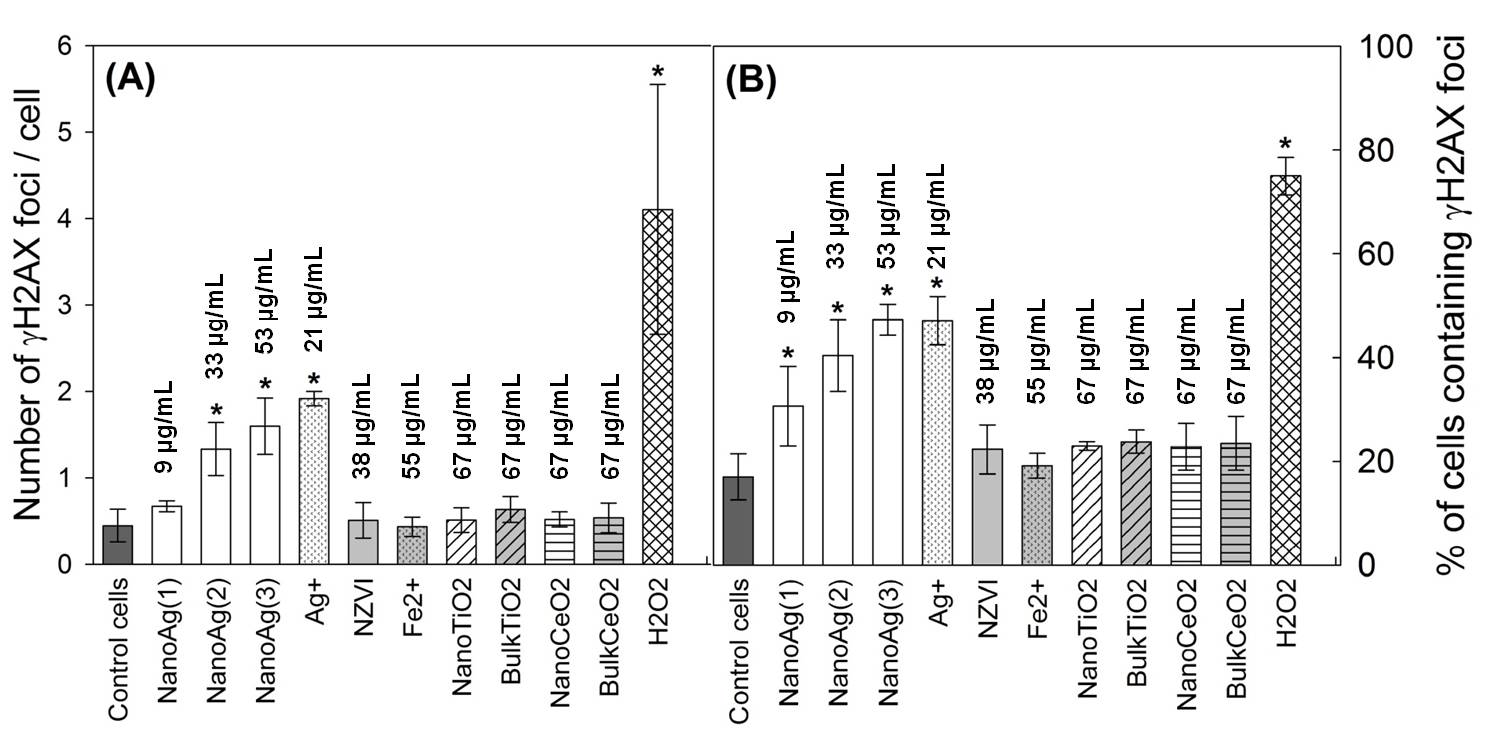


**Fig. S2**. γH2AX foci in untreated A549 control cells; cells treated with nanoAg, Ag^+^, NZVI, Fe^2+^, nanoTiO_2_, bulkTiO_2_, nanoCeO_2_, and bulkCeO2 at different concentrations for 24 h; and cells treated with 100 µM H_2_O_2_ for 10 min. Data are presented as (A) number of γH2AX foci per cell and (B) percentage of cells containing γH2AX foci. Error bars represent standard deviations of three independent experiments. “*” indicates significant difference compared with untreated control cells (p < 0.05).


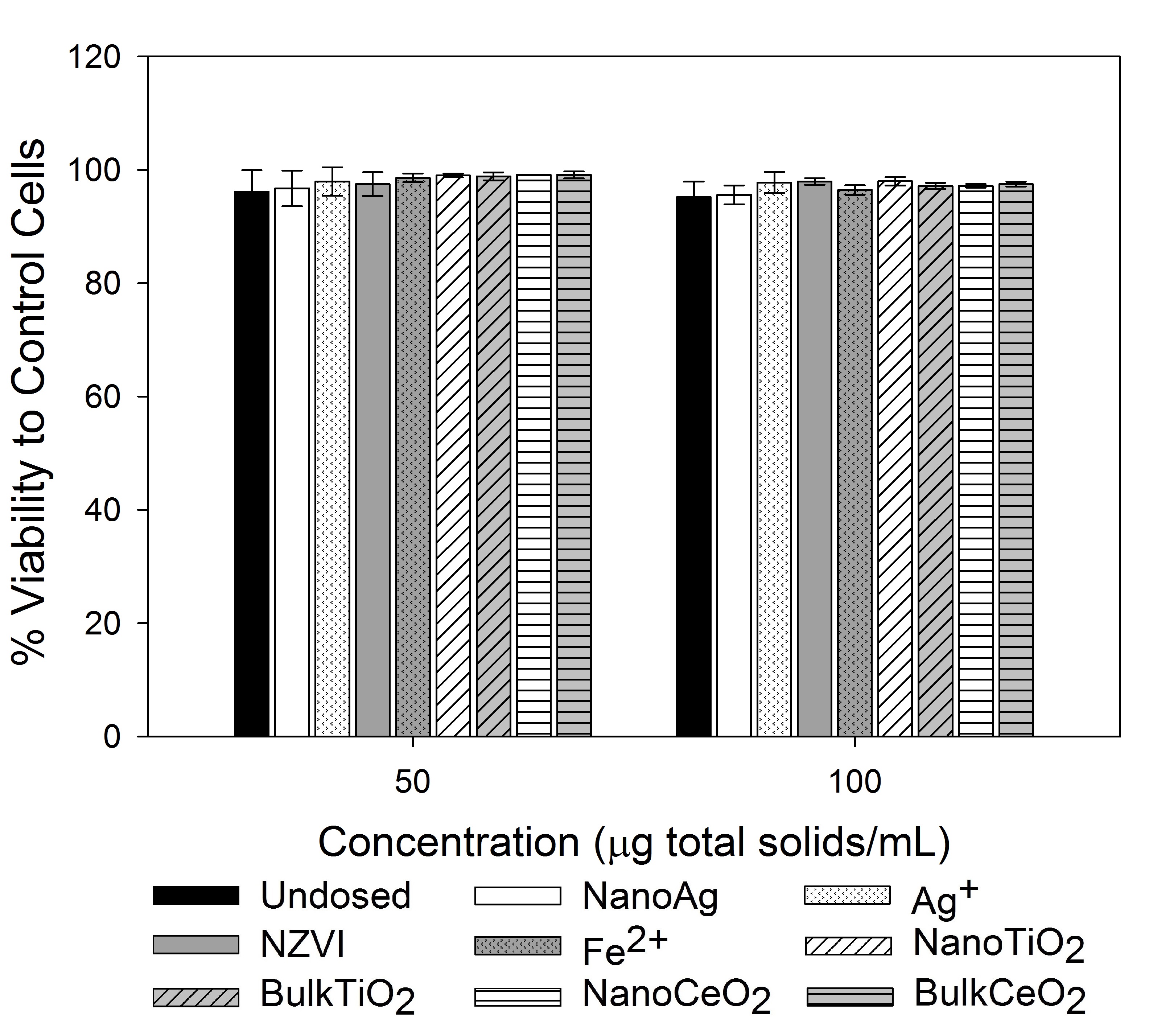


**Fig. S3.** Characteristic cytotoxicity of A549 cells exposed to 50 and 100 µg total solids/mL biosolids from undosed SBR, and SBRs dosed with nanoAg, Ag^+^, NZVI, Fe^2+^, nanoTiO_2_, bulkTiO_2_, nanoCeO_2_, and bulkCeO_2_ for 24 h by WST-1 assay. Error bars represent standard deviations of three independent experiments. There were no significant differences in viability between cells treated with biosolid samples compared with untreated control cells (p > 0.05).
